# The Role of Chronic Stress in Normal Visceroception: Insights From an Experimental Visceral Pain Study in Healthy Volunteers

**DOI:** 10.3389/fpsyt.2020.00107

**Published:** 2020-03-03

**Authors:** Adriane Icenhour, Franziska Labrenz, Till Roderigo, Sven Benson, Sigrid Elsenbruch

**Affiliations:** Institute of Medical Psychology and Behavioral Immunobiology, University Hospital Essen, University of Duisburg-Essen, Essen, Germany

**Keywords:** chronic stress, visceroception, gut-brain axis, visceral pain, urgency, recall bias, memory

## Abstract

Visceroception is a complex phenomenon comprising the sensation, interpretation, and integration of sensations along the gut-brain axis, including pain or defecatory urgency. Stress is considered a crucial risk factor for the development and maintenance of disorders of gut-brain signaling, which are characterized by altered visceroception. Although the broad role of stress and stress mediators in disturbed visceroception is widely acknowledged, the putative contribution of chronic stress to variations in normal visceroception remains incompletely understood. We aimed to elucidate the role of chronic stress in shaping different facets of visceroception. From a well-characterized, large sample of healthy men and women (N = 180, 50% female), volunteers presenting with low (n = 57) and elevated (n = 61) perceived chronic stress were identified based on the validated Trier Inventory for Chronic Stress (TICS). Visceral sensitivity together with perceived and recalled intensity and defecatory urgency induced by repeated rectal distensions was experimentally assessed, and compared between low and elevated stress groups. Subgroups were compared regarding state anxiety and salivary cortisol concentrations across experimental phases and with respect to psychological measures. Finally, in the full sample and in chronic stress subgroups, a recall bias in terms of a discrepancy between the perception of experimentally-induced symptoms and their recall was tested. Participants with elevated chronic stress presented with increased state anxiety and higher cortisol concentrations throughout the experimental phases compared to the group with low chronic stress. Group differences in visceral sensitivity were not evident. The elevated stress group perceived significantly higher urgency during the stimulation phase, and recalled substantially higher feelings of urgency induced by rectal distensions, while perceived and recalled intensity were comparable between groups. Volunteers with elevated stress exhibited a recall bias in terms of a higher recall relative to mean perception of urgency, whereas no such bias was observed for the intensity of experimental visceral stimulation. Our findings in healthy men and women provide first evidence that the troublesome symptom of urgency might be particularly modifiable by chronic stress and support the relevance of memory biases in visceroception. These results may help to disentangle the impact of chronic stress on altered visceroception in disturbances of gut-brain communication.

## Introduction

Visceroception is defined as the perception and processing of interoceptive signals arising from visceral organs (1, 2). Importantly, visceroception is not fully captured by visceral sensitivity alone, which primarily reflects sensory-discriminative aspects of perception. It is rather conceptualized more broadly as a specific facet of interoception, involving the sensation, interpretation, and integration of visceral signals (2) along the gut-brain axis. The complex affective-motivational and cognitive dimensions of visceroception shape gastrointestinal (GI) symptom perception, including visceral pain and defecatory urgency, in healthy individuals as well as in patients with chronic GI symptoms (3). The clinical relevance of disturbed visceroception is particularly evident in the context of disorders of disturbed gut-brain interactions, like irritable bowel syndrome (IBS) and functional dyspepsia. Altered GI symptoms perception, involving visceral hyperalgesia and hypervigilance, plays a pivotal role in their pathophysiology and treatment. However, the complex mechanisms underlying altered visceroception remain incompletely understood, especially with respect to psychological modulation.

As a crucial psychological factor, stress plays a broad role in disorders of disturbed gut-brain interactions (4–6). This has most clearly been underscored by evidence that acute stress or stress mediators of the hypothalamus-pituitary-adrenal (HPA) axis increase visceral sensitivity and neural processing of visceral stimuli in patients (7) but also in healthy volunteers (8). Chronic stress burden has been identified as an important risk factor for disease onset (9), and for the exacerbation of GI symptoms, particularly of visceral pain in patients with IBS (10, 11). Importantly, symptom burden in patients is often not limited to pain, but also involves defecatory urgency as a highly troublesome symptom in a broad range of GI conditions (12–14). Psychological modulation of defecatory urgency has previously been proposed (15), and our own recent data suggested that acute stress amplified nocebo effects especially for the symptom of distension-induced urgency more so then the symptom of pain in healthy volunteers (16). While together these findings clearly support a role of acute as well as chronic stress in different dimensions of visceroception, experimental work particularly on effects of chronic stress remains scarce.

Building on our earlier work on the modulation of visceroception by acute stress and stress mediators (7, 8, 16), we herein aimed to elucidate the putative role of chronic stress in different clinically-relevant facets of normal visceroception. From a large sample of well-characterized healthy men and women that underwent rectal sensitivity testing and repeated painful rectal distensions as part of a larger study (16, 17), we compared individuals with elevated and low perceived chronic stress with respect to sensory and pain thresholds and rectal distension-induced symptom reports of pain and urgency. We hypothesized that individuals with elevated levels of stress would reveal increased sensitivity, as reflected by lower thresholds for graded distensions of the rectum as well as higher pain and urgency ratings in response to individually-calibrated repeated distensions. In addition to analyses of symptom reports based on individual distensions, we also elucidated overall symptom recall based on a retrospective symptom rating. This was accomplished given evidence that retrospective overall symptom ratings may be more susceptible to psychological modulation, especially in patients with IBS (18). Given our interest in visceral pain-related memory effects (8, 19–22), together with evidence supporting the role of reporting bias in IBS (23), we introduced a new “memory bias” measure. This measure was based on the difference between perceptual ratings of individual distensions and retrospective overall ratings, the former being presumably more reflective of sensory-discriminative facts of visceroception, the latter possibly more prone to psychological modulation, both with relevance to collective symptom reporting in experimental and clinical trials as well as clinical practice.

## Methods

### Participants

From a well-characterized large sample of young healthy men and women (N = 180; 90 women), tertiles based on the validated Trier Inventory for Chronic Stress (TICS) were identified. The top and bottom tertile were chosen to define participants with low and elevated perceived chronic stress, as detailed below, and included in the current analysis. Participants had been recruited through local advertisements for the primary study on the modulation of placebo and nocebo effects by acute experimental stress (16) or relaxation (17) in visceral pain. They had been informed that the aim of the study would be to investigate psychological mechanisms underlying effects of different drugs on experimentally-induced visceral symptoms. Of note, all measures included in the current analyses were assessed prior to randomization of participants for subsequent experimental manipulations. They served as baseline measures in the primary studies, and observations reported herein were therefore independent of subsequent manipulations. Participants were excluded according to the following criteria: age < 18 or > 65 years, body mass index (BMI) < 18 or > 30, a history of or acute medical and psychiatric conditions and current medication use except for hormonal contraceptives, thyroid medication, and occasional use of over-the-counter pain or allergy medication. Moreover, subclinical gastrointestinal (GI) symptoms experienced during a 3 month period preceding study participation were measured using a standardized in-house questionnaire (24). As in our prior studies (19, 21, 24) a cut-off score of 11 was used as an indicator of a putative undiagnosed gastrointestinal condition, which led to exclusion from study participation. Only women using hormonal contraception were included and pregnancy was ruled out on the study day with a commercially available urinary test. All participants underwent a physical examination to exclude perianal tissue damage (e.g., fissures or painful hemorrhoids), which might interfere with the experimental procedure. Participants gave written informed consent and received 200€ for their participation. The study protocol was approved by the local ethics committee (protocol number 13-5565-BO) and followed the provisions of the Declaration of Helsinki.

### Experimental Design and Study Procedures

**Figure 1:**
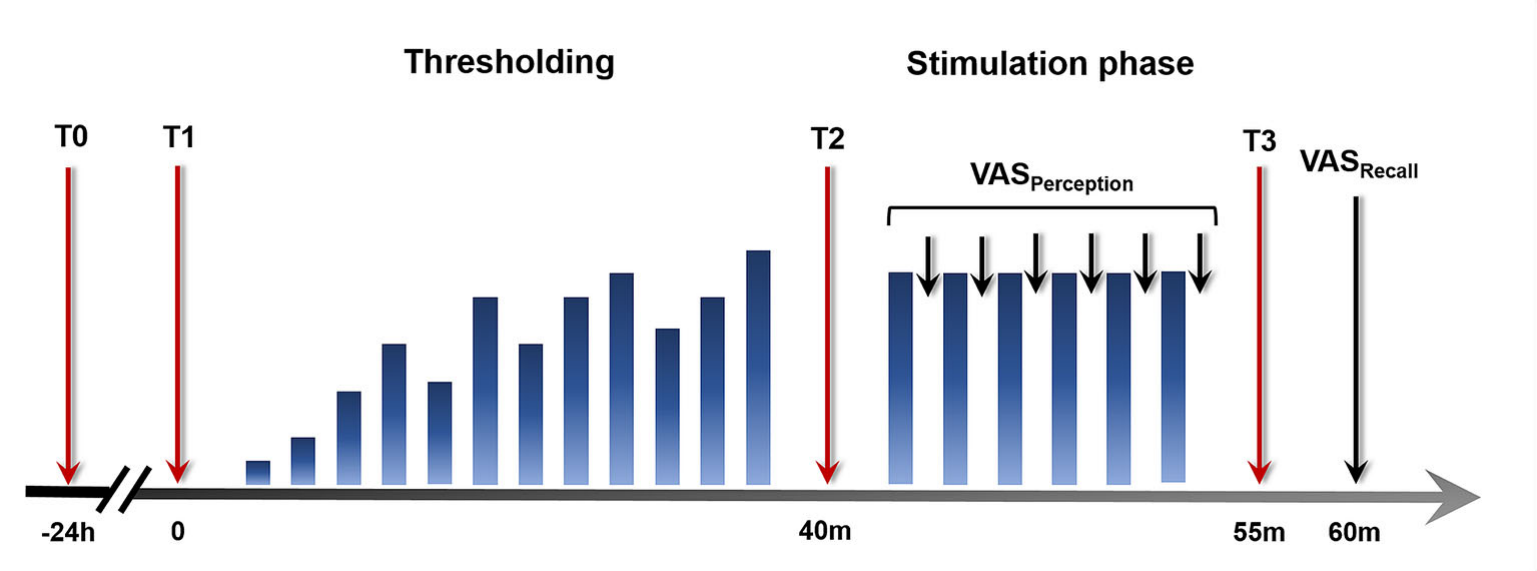
Study design and experimental procedures. Twenty-four hours prior to study participation (T0), a salivary cortisol sample was collected as a baseline measure unaffected by the experimental procedure. On the study day, salivary cortisol and state anxiety were assessed upon arrival (T1), before (T2), and after (T3) the stimulation phase. Visual analog scale (VAS) ratings of intensity and urgency perception were acquired during the stimulation phase. At the conclusion of the experimental phase, VAS ratings assessing intensity and urgency recall were accomplished.

An overview over the experimental design and the study procedures relevant to the current analyses is given in **Figure 1**. All experimental procedures were conducted between 12:00 and 18:00 h to account for effects of the circadian rhythm. Initially, an inflatable balloon attached to a pressure-controlled barostat system (modified ISOBAR 3 device; G & J Electronics, ON, Toronto, Canada) was placed 5 cm from the anal verge, for the application of rectal distensions. Rectal sensory and pain thresholds were determined using a double-random staircase distension protocol with random pressure increments between 2 and 6 mmHg and a maximal distension pressure of 55 mmHg. Participants rated each sensation on a Likert-type scale labeled 1 = no perception, 2 = doubtful perception, 3 = sure perception, 4 = little discomfort, 5 = severe discomfort, and 6 = pain, not tolerable distension. The sensory threshold was defined as a pressure when ratings changed from 2 to 3 and the pain threshold was determined at the change from 5 to 6. The individual pain threshold was used as an anchor for a subsequent pressure calibration to identify a moderately painful intensity for the repeated application of rectal distensions. Specifically, a pressure corresponding to a pain intensity rating not higher than 80 on a visual analog scale (VAS) with endpoints labeled 0 = none at all and 100 = very much was identified, as previously described (16). This intensity was used for the subsequent stimulation phase, during which six rectal distensions with a duration of 30 s and a rest interval of 30 s following each stimulus were applied. Salivary cortisol concentrations and state anxiety as measures of acute stress and arousal were collected at different time points across the experimental phases and ratings of stimulus intensity and urgency perception and recall as different facets of visceroception were assessed, as detailed below.

### Measures of Visceroception

In addition to sensory and pain thresholds, mean scores of trial-by-trial VAS ratings of stimulus intensity and urgency perception and ratings of overall recalled intensity and urgency induced by the experienced rectal distensions during the stimulation phase were assessed as measures of visceroception. Specifically, during the stimulation phase, participants were prompted to rate the intensity of each distension and the urgency it induces on separate VAS with endpoints labeled “none” (0) and “very much” (100) for intensity and “none (0) and “very high” (100) for urgency. Following the stimulation phase, participants were asked to recall the overall intensity of and urgency induced by the experienced distensions using separate VAS. In order to elucidate a potential memory bias in visceroception in terms of a deviation of recalled from mean perceived visceral sensations, delta values between perceived and recalled intensity and urgency were calculated, respectively, to test for recall biases in measures of visceroception.

### Assessment of State Anxiety and Salivary Cortisol

Twenty-four hours prior to the study appointment (time point T0), a baseline salivary cortisol sample was collected by participants in their home environment, using Salivettes (Sarstedt, Nürnbrecht, Germany), and stored at 4°C until transport to the laboratory on the study day. On the study day, salivary cortisol as a marker of acute stress and HPA axis activation was collected upon arrival (T1), following the thresholding procedure before the stimulation phase (T2), and after the stimulation phase (T3). As a self-report measure of acute arousal, state anxiety was assessed at time points T1–T3 along with cortisol sample collection, using the state version of the validated State Trait Anxiety Inventory (STAI-S) (25, 26). Saliva samples were centrifuged (2,000 rpm, 2 min, 4°C) and stored at −20°C until analysis. Cortisol concentrations were measured using an enzyme-linked immunosorbent assay (ELISA; IBL International, Hamburg, Germany) in accordance with the manufacturer’s protocol with a detection limit at 0.138 nmol/L.

### Assessment of Chronic Stress and Identification of Stress Subgroups

Following informed consent, participants completed the validated Trier Inventory for Chronic Stress (TICS) screening scale (27). The self-assessment instrument allows an evaluation of individual experiences with chronic stressors in everyday life, providing a reliable global measure of perceived stress during the previous 3 months with a Cronbach’s α of .91 (28). Each of the 12 items is scored on a five-point Likert-scale as “never” (0), “rarely” (1), “sometimes” (2), “often” (3), and “very often” (4 points). The total score ranges from 0 to 48 points, expressing the subjectively perceived presence and frequency of chronic stressors. Norm values from healthy volunteers are available (22), with a mean TICS score of 13 corresponding to T = 50. TICS sum scores were used herein to evaluate overall perceived chronic stress and to allocate participants to a subgroup with low or elevated chronic stress. This was accomplished by subdividing participants into tertile subgroups based on TICS scores. Participants in the top tertile were defined as an elevated stress subgroup, the bottom tertile as a group with low chronic stress.

### Questionnaires

In addition to TICS for the assessment of chronic stress, participants completed the following comprehensive questionnaire battery for a characterization with respect to psychological factors of putative relevance to both, stress, and visceroception: The trait version of the State Trait Anxiety Inventory (STAI-T) (25, 26) for the assessment of trait anxiety (sum scores between 20 and 80), the Pain-Related Self Statements Scale (PRSS) (29) to measure pain-related cognitions in terms of maladaptive pain catastrophizing and adaptive pain coping (sum scores ranging from 0 to 45, respectively), and the Generalized Self-efficacy (GSE) Scale (30) to assess optimistic self-beliefs to cope with life demands (total scores 10–40) as a marker of resilience to stressors.

### Statistical Analyses

All statistical analyses were performed using IBM SPSS version 25 (IBM Corporation, Armonk, NY, USA). As described above, participants were stratified based on the level of perceived chronic stress, allowing to define and compare groups with low and elevated perceived chronic stress. Notably, due to this stratification strategy, the investigated samples displayed non-normal distribution in some of the relevant outcome measures, as evidenced by significant Kolmogorov-Smirnov tests. However, no outliers were detected in either sample. Given sufficient sample sizes and the robustness of parametric statistical approaches under these circumstances, parametric tests were performed. Accordingly, stress subgroups were compared with respect to sociodemographic and psychological characteristics using two sample t-tests or chi square test where appropriate. Group comparisons of sensory and pain thresholds as measures of visceral sensitivity, as well as baseline cortisol (T0) were accomplished using two sample t-tests. Repeated measures ANOVA with the within-group factor *time* and the between-group factor *stress subgroup* were applied to analyze state anxiety and salivary cortisol concentrations on the study day (T1–T3). Independent sample t-tests were further conducted for group comparisons of mean perceived and recalled measures of visceroception, as assessed with VAS. In addition, bias scores based on the difference between perceptual and retrospective ratings were entered into one sample t-tests for effects in the full sample and into two sample t-tests for stress subgroup comparisons. To account for a possible impact of acute stress and arousal on effects of chronic stress on visceroception, analyses of covariance (ANCOVA) with mean cortisol concentrations and mean state anxiety scores as covariates were additionally conducted for measures of visceroception. Further, to address possibly divergent effects of chronic stress on measures of visceroception in men and women, interactions between the factors *sex* and *stress subgroup* were explored using ANOVA. Finally, to confirm the specificity of findings to chronic stress, stepwise multiple regression analyses (probability to enter ≤.05, probability to remove ≥.10) were performed in the full sample, entering TICS scores as a measure of perceived chronic stress together with closely related psychological traits, such as trait anxiety, pain coping, and self-efficacy, as predictors of variance in visceroceptive markers. Results from ANOVA and ANCOVA are reported with Greenhouse-Geisser correction to account for a possible violation of the sphericity assumption and results from *post hoc* t-tests were Bonferroni corrected for multiple comparisons where appropriate. Alpha level was set at *p* < .05, exact two-tailed *p* values are reported and *η_p_^2^*, Cohen’s *d*, or Cramer’s *V* are provided as indicators of effect size, respectively. All descriptive statistics are reported as mean ± standard error of the mean (SEM), unless indicated otherwise.

## Results

### Sample Characterization

A characterization of the full sample and comparisons of stress subgroups with respect to sociodemographic and psychological measures are summarized in **Table 1**. TICS scores in the full sample indicated an average level of perceived chronic stress according to available norm values (28). The mean score in the low stress group corresponded to an average level of chronic stress within a lower range in a healthy population. The elevated stress group presented with mean TICS scores above average, confirming the stratification strategy and the identification of healthy volunteers with low and elevated levels of perceived chronic stress. Accordingly, chronic stress scores were substantially higher in the elevated chronic stress group. Subgroups were comparable regarding age, BMI, and distribution of men and women. Participants with elevated stress presented with increased trait anxiety, lower self-efficacy, and reported more catastrophizing cognitions when coping with pain, whereas groups did not differ regarding the use of active pain coping.

**Table 1:** Characterization of the full sample and chronic stress groups with respect to sociodemographic and psychological variables.

	Full sample N = 180	Low stress n = 57	Elevated stress n = 61	t/*χ* ^2^	p	*d/V*
**Female (n, %)**	90 (50%)	29 (50.9%)	29 (47.5%)	0.13	.717	.033
**Age**	26.38 ± 0.45	27.23 ± 0.92	26.43 ± 0.82	0.65	.516	.012
**BMI**	23.29 ± 0.21	23.40 ± 0.36	23.67 ± 0.41	0.49	.623	.009
**Chronic stress (T)**	17.58 ± 0.65 (55)	7.93 ± 0.49 (44)	26.98 ± 0.64 (63)	23.68	**<.001**	.434
**Trait anxiety**	36.03 ± 0.64	29.81 ± 0.67	43.03 ± 1.14	9.98	**<.001**	.182
**Pain catastrophizing**	1.88 ± 0.06	1.45 ± 0.09	2.45 ± 0.11	6.96	**<.001**	.128
**Active pain coping**	3.41 ± 0.06	3.46 ± 0.11	3.36 ± 0.10	0.74	.460	.014
**Self-efficacy**	30.25 ± 0.30	32.14 ± 0.44	28.00 ± 0.53	6.01	**<.001**	.111

Data are given as mean ± SEM, unless indicated otherwise and significant group differences are indicated in bold. BMI, body mass index; T, T-score for TICS screening scale.

### Salivary Cortisol and State Anxiety

**Figure 2:**
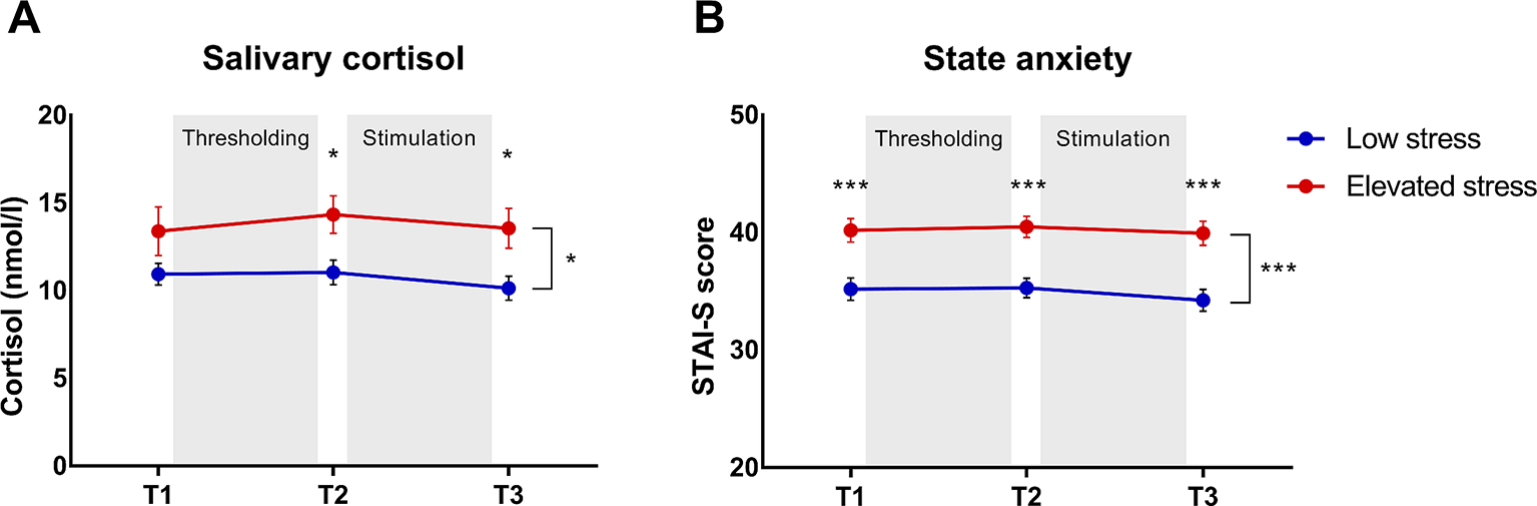
Group comparisons of **(A)** salivary cortisol concentrations and **(B)** state anxiety across the experimental phases (T1–T3) in subjects with elevated (n = 61) *versus* those with low chronic stress (n = 57). Data are given as mean ± SEM. *p < .05 ***p < .001.

Baseline cortisol concentrations 24 h prior to the study appointment (T0) were significantly increased in the group with elevated chronic stress (13.29 ± 1.24 nmol/l) relative to individuals with low perceived chronic stress (10.09 ± 0.72 nmol/l; *t* = 2.24; *p* = .027; *d* = .042). Analysis of cortisol concentrations on the study day (T1–T3) revealed a significant effect of *stress subgroup* (*F* = 6.60; *p* = .011; *η_p_^2^* = .054), which was attributable to higher cortisol levels across the experimental phases in participants with elevated perceived chronic stress (**Figure 2A**). No effect of *time* was evident (*p* = .372). Analysis of state anxiety also demonstrated a significant effect of *stress subgroup* (*F* = 19.76; *p* < .001; *η_p_^2^* = .146), with higher state anxiety in the elevated stress compared to the low stress group (**Figure 2B**). No effect of *time* was observed (*p* = .257).

### Visceroception in Subgroups With Low and Elevated Chronic Stress

#### Sensory and Pain Thresholds

**Figure 3:**
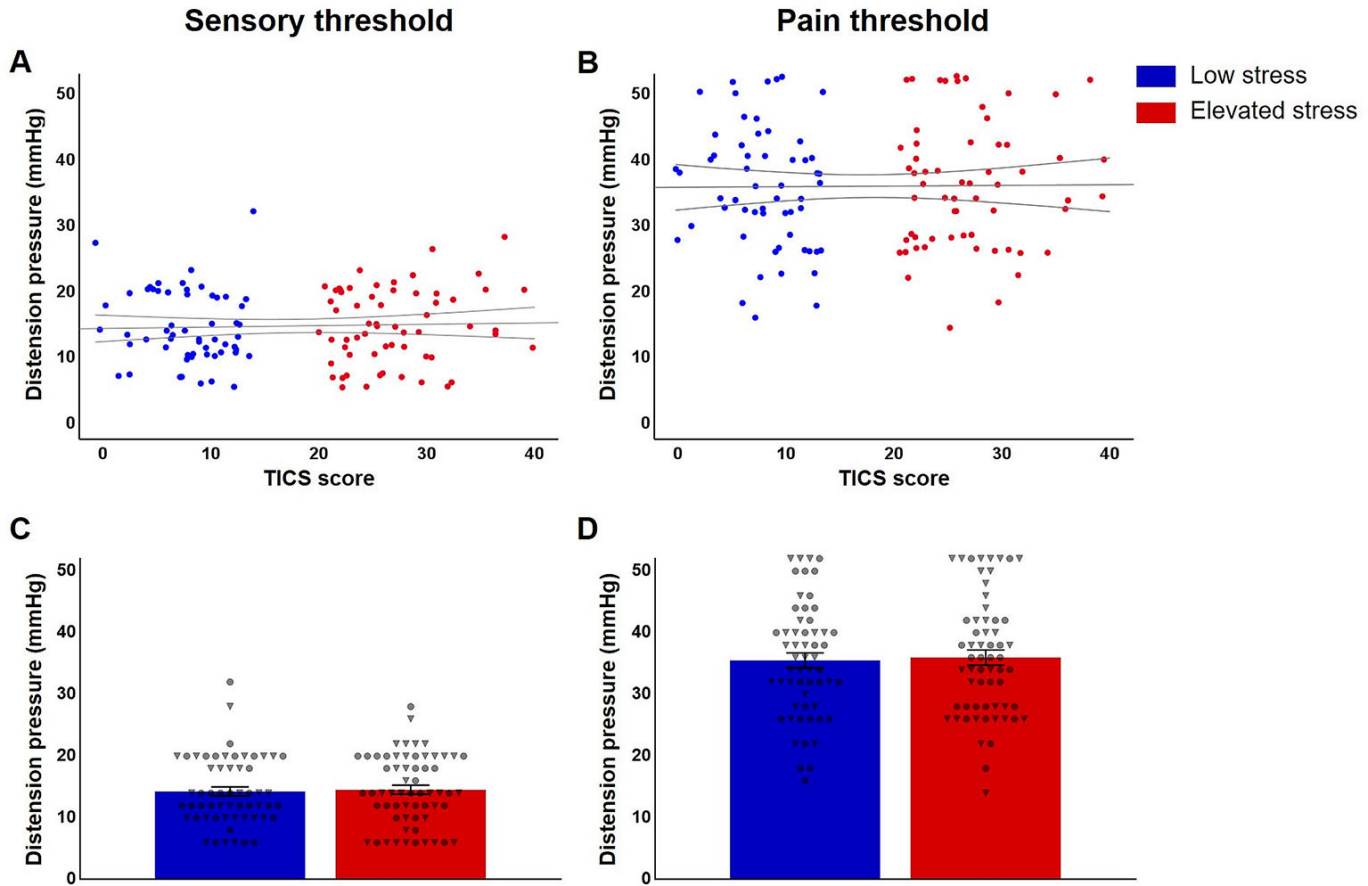
Jittered scatterplots with regression curves and 95% confidence intervals of individual **(A)** sensory and **(B)** pain thresholds in participants with low (n = 57, blue) and elevated chronic stress (n = 61, red) and group comparisons regarding mean thresholds **(C, D)**, provided ± SEM and depicted with individual data points for women (indicated as circles) and men (shown as triangles).

Analyses of sensory and pain thresholds in individuals with elevated and low perceived chronic stress revealed comparable thresholds for both, first sensation (*p* = .789; **Figures 3A, C**) and pain (*p* = .794; **Figures 3B, D**). Controlling for state anxiety and salivary cortisol concentrations did not affect these results (data not shown).

#### Perception and Recall of Visceroceptive Stimulation

**Figure 4:**
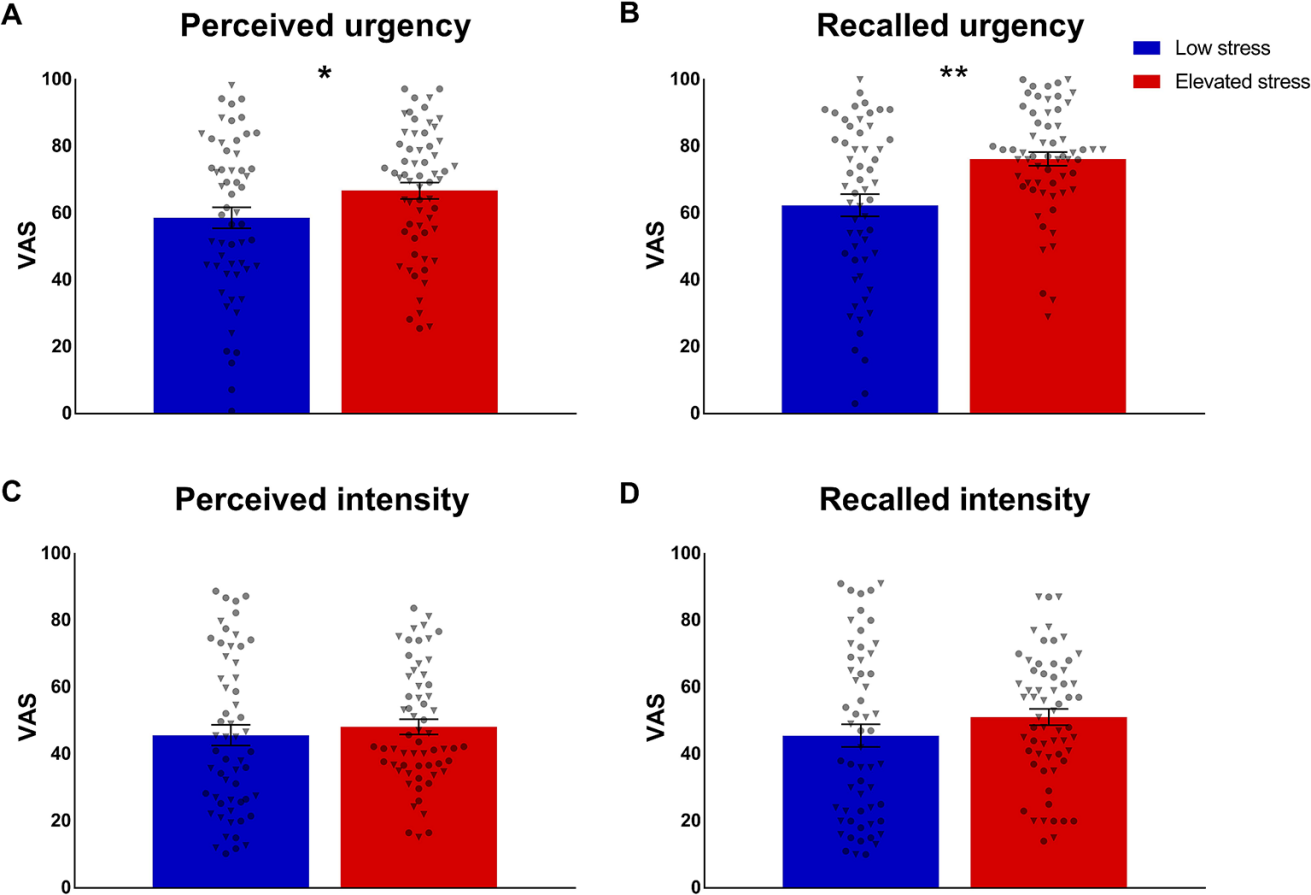
Group comparisons of **(A)** mean perception and **(B)** recall of urgency and **(C, D)** intensity of repeated rectal distensions during the stimulation phase in participants with low (n = 57) and elevated (n = 61) chronic stress. Data are given as mean ± SEM and individual data points for women (circles) and men (triangles) are provided. *p < .05; **p < .01.

Subgroup comparisons of mean perceived urgency based on trial-by-trial ratings following each visceral sensation during the stimulation phase revealed significantly higher urgency in the group with elevated stress (*t* = 2.04; *p* = .043; *d* = 0.37; **Figure 4A**), whereas groups did not differ regarding mean perceived intensity (*p* = .507; **Figure 4C**). Similarly, participants with elevated perceived chronic stress recalled significantly higher overall urgency experienced during the stimulation phase (*t* = 3.57; *p* = .001; *d* = 0.66; **Figure 4B**), but no group difference in recalled intensity was observed (*p* = .517; **Figure 4D**). In covariance analyses, group differences in mean perceived urgency failed statistical significance (*p* = .095) when including mean state anxiety and mean cortisol concentrations, while differences in urgency recall remained widely unaffected (*F* = 9.44; *p* = .003; *η_p_^2^* = .076) and no changes were evident regarding perceived or recalled intensity (data not shown).

#### Recall Bias

**Figure 5:**
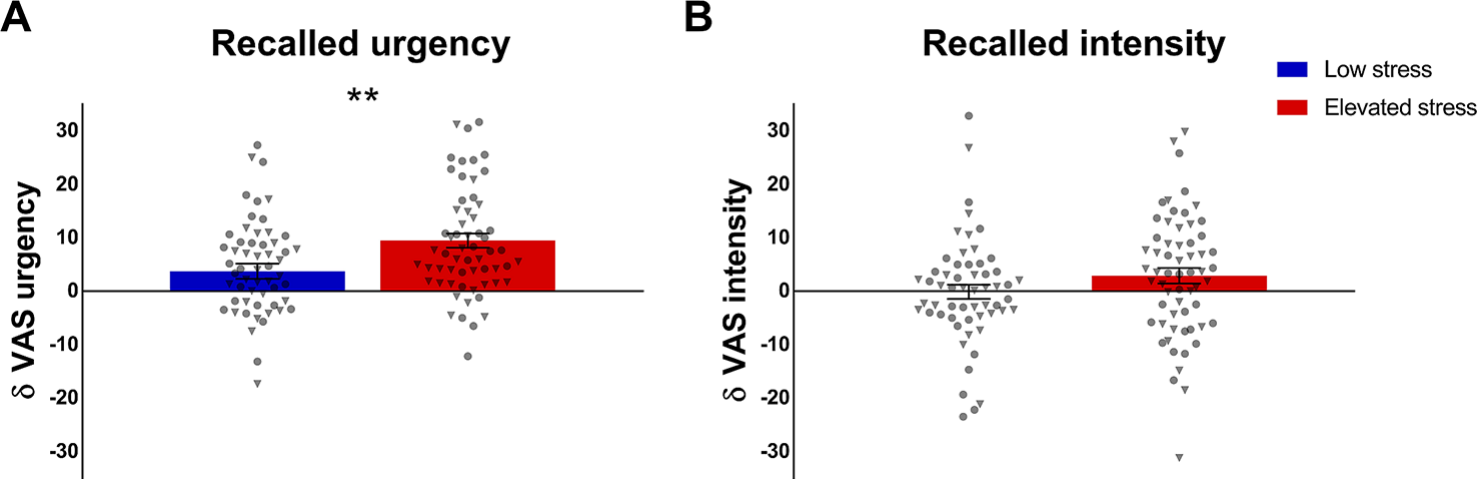
Group comparisons in recall bias of **(A)** defecatory urgency and **(B)** intensity of rectal distensions during the stimulation phase, operationalized as the difference between mean perceived and recalled symptoms. Data from individuals with low (n = 57) and elevated chronic stress levels (n = 61) are given as mean ± SEM and individual data points are illustrated as circles for women and as triangles for men. **p < .01.

To elucidate a putative exaggeration of intensity or urgency recall, the full sample and stress subgroups were tested for a recall bias in visceroception, operationalized as the differences between mean reported perception during the stimulation phase and overall recall, respectively. One sample t-tests revealed significant effects for both, intensity (*t* = 2.48; *p* = .014; *d* = 0.18) and, more pronounced, for urgency (*t* = 8.38; *p* < .001; *d* = 0.62), indicating higher recall relative to mean perception in the full sample. Individuals with elevated chronic stress exhibited a significant bias for recalled defecatory urgency, i.e., recalled more intense feelings of urgency relative to their mean perception (*t* = 2.96; *p* = .004; *d* = 0.55; **Figure 5A**). The recall bias for intensity was comparable between stress subgroups (*p* = .132; **Figure 5B**). ANCOVA including mean state anxiety and cortisol did not affect these finding (urgency recall bias: *F* = 6.68; *p* = .011; *η_p_^2^* = .055; intensity recall bias: *p* = .305).

### Interactions Between Chronic Stress and Sex

Possible sex differences in the effects of perceived chronic stress on visceroception were addressed in exploratory analyses. For thresholds, results revealed no interaction between stress level and sex for either first sensation (*p* = .950; **Figure 3C**) or pain (*p* = .451; **Figure 3D**). No evidence of sex-specific effects of chronic stress emerged for perceived (*p* = .503; **Figure 4A**) and recalled urgency (*p* = .824; **Figure 4B**) or intensity (perceived: *p* = .143; **Figure 4C**; recalled: *p* = .222; **Figure 4D**). Finally, neither urgency (*p* = .352; **Figure 5A**) nor intensity recall bias (*p* = .793; **Figure 5B**) indicated sex-specific effects of perceived chronic stress.

### Specificity to Chronic Stress

Stepwise multiple regression analyses were performed in the full sample of N = 180 participants to evaluate whether the observed effects were specific to chronic stress or could also be attributed to effects of other psychological traits, including trait anxiety, pain coping strategies, and self-efficacy. This exploratory approach focused on significant findings from subgroup analyses, and was therefore conducted on perceived and recalled urgency as well as the urgency recall bias. These analyses confirmed chronic stress to be the main predictor of visceroception. Specifically, TICS scores were a single significant predictor of mean perceived (*F* = 4.49; *p* = 0.035; *adj. R^2^* = 0.018; *ß* = 0.157) and, more pronounced, recalled urgency (*F* = 12.84; *p* < .001; *adj. R^2^* = 0.062; *ß* = 0.259). Further, perceived chronic stress was identified as a single predictor of variance in the urgency recall bias (*F* = 8.59; *p* = .004; *adj. R^2^* = 0.041; *ß* = 0.215). Trait anxiety, pain coping, and self-efficacy did not contribute additionally to explaining variance in visceroception.

## Discussion

The relevance of interoception for both health and disease is increasingly acknowledged (1, 2, 31), especially in the context of visceral hypersensitivity in disorders of gut-brain interactions. Although the broad role of stress and stress mediators in disturbed visceroception is widely appreciated (32–34), the putative contribution of chronic stress to variations in normal visceroception remains incompletely understood. To fill this research gap, we herein assessed the impact of chronic stress on different dimensions of visceroception induced by rectal distensions by comparing stress subgroups classified based on a validated chronic stress questionnaire. While individuals with elevated *versus* low levels of perceived chronic stress did not differ in rectal sensory or pain thresholds, both the perception as well as the recall of rectal urgency were significantly enhanced in individuals with elevated chronic stress. Furthermore, a recall bias for previously experienced distension-induced urgency was more pronounced in the group with elevated stress. Together, these findings support that the sensation of urgency might be particularly modifiable by chronic stress in healthy young men and women, with implications for the pathophysiology of chronic GI symptoms.

A link between chronic stress and the perception and recall of urgency complements our previous result that the symptom of urgency was demonstrably highly modifiable by acute psychosocial stress in a placebo/nocebo paradigm (16). Psychological modulation of urgency is interesting from a clinical perspective for a broad range of conditions characterized by chronic gastrointestinal symptoms, which are often not limited to the experience of visceral pain. For example, defecatory urgency is a symptom frequently reported by patients with IBS (12), which has recently been identified as the most troublesome symptom in diarrhea-predominant IBS (13). Urgency has also emerged as an independent predictor of quality of life not only in IBS and other disturbances of gut-brain communication (35, 36), but also in the general population (37).

In light of the fact that symptom reports guide diagnosis and treatment in many conditions involving the gut-brain axis, our findings suggesting a putative role of chronic stress in GI symptom recall are noteworthy and deserve more attention. The recall of defecatory urgency induced by previously experienced visceral sensations was enhanced in participants with elevated chronic stress. Further, individuals who reported more chronic stress also demonstrated a more pronounced recall bias for urgency, herein quantified as the difference between the individual distension ratings and the overall urgency recall. The role of reporting bias and its possible contribution to findings of visceral hypersensitivity in IBS has previously been elegantly demonstrated (23). Our results expand on these data using a somewhat simpler yet clinically-relevant method, following a line of research on memory processes in visceroception (8, 19–22), with a particular focus on interoceptive hypervigilance. It is indeed intriguing to speculate that chronic stress may contribute to interoceptive hypervigilance, either indirectly involving a reporting bias or more directly by biasing specific memory processes, including immediate recall, toward more “negative” memories of symptoms. Future studies should therefore test the hypothesis that altered visceroceptive recall may constitute a nocebo mechanism in the pathophysiology of altered gut-brain interactions. Support for this assumption is provided by experimental findings from the field of associative visceral pain-related conditioning (19, 21, 22), with documented alterations in pain-related learning and memory processes in patients with IBS (20, 38). Stress and stress mediators might play a key role in these alterations, as evidenced by findings that antagonizing corticotropin releasing factor, one of the main signaling peptides of the HPA axis released in response to stress, normalized aberrant neural and psychophysiological correlates of abdominal pain-related learning and memory in women with IBS (38). In healthy individuals, we recently observed pharmacologically increased cortisol levels to induce a reduction in visceral pain thresholds and to affect the formation of pain-related emotional memories (8). Importantly, these effects appeared to be specific to the visceral domain and were not observed for somatic stimuli of identical intensities, in line with prior research on distinct mechanisms underlying the processing of visceral and somatic pain (39–42), and suggesting that visceroception might be particularly vulnerable to stress and stress mediators. Our findings expand this evidence to the dimension of chronic stress, with putative clinical implications for vulnerability and resilience in health and in disorders of gut-brain communication.

On a critical note, some of the mechanisms underlying our findings remain difficult to discern. We observed significantly elevated state anxiety and cortisol concentrations in our cohort of individuals with higher perceived chronic stress across experimental time points. Hence, higher perceived chronic stress was clearly associated with differences in “state” measures, which reportedly modulate visceral pain processing (43), but also with psychological traits, such as increased trait anxiety, maladaptive pain coping, and lower self-efficacy. While our explorative covariance and regression analyses widely supported the observed effects to be distinctly attributable to chronic stress, our study design and the present results do not allow conclusive answers. Clearly, there exists a large overlap between chronic stress and trait anxiety, including its underlying neurobiology (44) and maladaptive coping is likely to further increase not only acute stress responsivity but also the burden arising from physical or psychological stressors (45). These psychological factors might therefore further increase detrimental effects of chronic stress in patients with disorders of gut-brain interactions. Future studies may consider including patients with disturbed visceroception with and without a comorbidity with anxiety. This could shed more light on additive or interactive relations between stress and anxiety in visceroception, which cannot be fully captured in our sample of young, healthy individuals with overall low anxiety symptom burden and adaptive coping skills. Likewise, although we identified a group of healthy individuals in our sample reporting levels of chronic stress above average, the stress burden was not substantially increased to clinically-relevant levels (20, 46). While posing a limitation regarding the generalizability of our findings to the impact of severe chronic stress in patients with disturbed visceroception, our data support that even subtle increases in perceived chronic stress might modulate visceroception. Finally, we addressed our research questions regarding the role of chronic stress in visceroception in a mixed sample of men and women. A recent analysis conducted in a large pooled sample including the current study cohort confirmed no differences between healthy male and female participants with respect to visceral sensitivity, yet did not test other aspects of visceroception (47). Stress subgroups in the current sample did not differ in the relation between male and female participants, suggesting that men and women suffered from elevated chronic stress to comparable extents. Further, exploratory analyses indicated no sex- or gender-specific effects of perceived chronic stress on the tested dimensions of visceroception, suggesting that, at least in young healthy men and women, chronic stress and sex/gender do not interact in altering visceroception. On the other hand, pharmacologically increased cortisol was recently shown to affect visceral sensitivity distinctly in women (8), in support of a role of sex/gender in the impact of the stress mediator on visceroception. Importantly, such putative sex-dependent effects of stress or stress markers might be mediated by interactions with gonadal hormone status, which in women is subject to substantial variations across the menstrual cycle, reportedly impacts visceroception (48), and appears to affect the vulnerability and responsivity to stressors (49, 50). To control for confounding effects of menstrual cycle phase, all women in the sample under investigation were on hormonal contraception. However, this selection does not allow a generalization to women with a natural menstrual cycle, calling for future research including female participants in different mentrual cycle phases.

Taken together, our findings support that elevated perceived chronic stress affects visceroception in healthy individuals, particularly the perception and recall of defecatory urgency as a highly disturbing and clinically-relevant marker in patients with disturbances of gut-brain interactions (35, 36). As two major pathways in the communication along the gut-brain axis, the descending stress system and the ascending visceroceptive system are tightly interacting (32) and their dysfunction may have profound detrimental effects on the communication pathways connecting the brain and the gut. Therefore, investigating chronic stress also in otherwise healthy individuals may aid to gain further insights into mechanisms contributing to long-lasting disturbances along the gut-brain axis. Importantly, the relevance of a dysfunctional gut-brain interaction is increasingly acknowledged beyond disorders primarily characterized by GI symptoms. These developments are not least owed to a growing appreciation of the crucial role of gut microbiota in health and disease (51–53). Particularly, tremendous advances have been made in understanding the impact of pre- and postnatal microbial composition on responsivity to stress later in life (54). These transdisciplinary findings strongly suggest a key role of the microbiota-gut-brain axis and its neural, humoral, endocrine, and immunological communication pathways in stress-related disturbances. This has implications for both the pathophysiology, and also the therapy of diseases affecting visceroception (6, 55), as well as highly comorbid stress-related central nervous system (CNS) disorders (56), such as anxiety (57), depression (58), and posttraumatic stress disorder (PTSD) (59). Interdisciplinary research on the complex communication pathways along the gut-brain axis bridging neurogastroenterology, psychiatry, and the neurosciences therefore promises important new insights into pathophysiological processes and may inspire new treatments of diseases characterized by altered stress responsivity, including those with visceroceptive malfunction.

## Data Availability Statement

The datasets generated for this study are available on request to the corresponding author.

## Ethics Statement

The studies involving human participants were reviewed and approved by the Institutional Ethics Review Board of the Medical Faculty of the University of Duisburg-Essen. The patients/participants provided their written informed consent to participate in this study.

## Author Contributions

SE and SB designed the research study. TR performed the research. AI and FL analyzed the data. AI, SB, and SE wrote the paper. All authors revised the manuscript for critical intellectual content and approved the final version of the manuscript.

## Funding

This project was funded by the Deutsche Forschungsgemeinschaft (DFG, German Research Foundation) as part of the DFG Research Unit FOR 1328 (grant number: EL 236/8-2) and project number 316803389 – SFB 1280. The funding agency had no role in the conception, analysis, or interpretation of the data.

## Conflict of Interest

The authors declare that the research was conducted in the absence of any commercial or financial relationships that could be construed as a potential conflict of interest.
